# Supplementary material for: Thermodynamic Driving Forces for Substrate Atom Extraction by Adsorption of Strong Electron Acceptor Molecules
Source: J Phys Chem C Nanomater Interfaces. 2022 Mar 28;126(13):6082–90. doi: 10.1021/acs.jpcc.2c00711 (PMC9007530; doi:10.1021/acs.jpcc.2c00711)
Supplement: Supplementary file 1 — jp2c00711_si_001.pdf [file jp2c00711_si_001.pdf]

Supporting Information to

Thermodynamic Driving Forces for Substrate Atom Extraction by Adsorption of Strong Electron Acceptor Molecules

Paul Ryan[1,2], Philip James Blowey[1,3], Billal S. Sohail[4], Luke A. Rochford[1], David A. Duncan[1], Tien-Lin Lee[1], Peter Starrs [1, 5], Giovanni Costantini[4], Reinhard J. Maurer[4*] David Phillip Woodruff[3*]

(1) Diamond Light Source, Harwell Science and Innovation Campus, Didcot, OX11 0DE, UK

(2) Department of Materials, Imperial College, London SW7 2AZ, UK

(3) Department of Physics, University of Warwick, Coventry CV4 7AL, UK

(4) Department of Chemistry, University of Warwick, Coventry CV4 7AL, UK

(5) School of Chemistry, University of St. Andrews, St. Andrews, KY16 9AJ, UK

Contents

1. Additional experimental results: LEED, STM, SXPS and NIXSW
2. Additional DFT structural and energetics results: TCNQ and F_4TCNQ on Ag(100)

1. Additional experimental results: LEED, SXPS and NIXSW

STM images and LEED patterns recorded from all of the ordered phases of TCNQ and F_4-TCNQ on Ag(100) are shown in Figure S1, together with simulations using the LEEDpat program[1] based on the matrices reported in Table S1. The LEED patterns observed from surfaces containing the two coexisting windmill structures (TCNQ:W1 and TCNQ:W2) were complex, as may be expected from the coexistence of two such large unit mesh structures, so no attempt has been made to identify all the observed diffracted beams, but an example of the diffraction pattern is shown in Figure S1. Notice that an unusual feature of the LEED pattern from the F_4TCNQ overlayer is the splitting into groups of four spots of all beams with indices comprising one integer and one half-integer, such as (0 ½), (-1 -½) (but not (½ ½)). These beams are circled in Figure S1. The splitting is not reproduced in the simulated pattern using the commensurate matrix $\begin{pmatrix} 2 & -2 \\ 4 & 2 \end{pmatrix}$ and is attributable to antiphase domain boundaries on the overlayer structure. This assignment is supported by the observation that the splitting was found to gradually disappear with extended annealing.

* corresponding authors d.p.woodruff@warwick.ac.uk, r.maurer@warwick.ac.uk

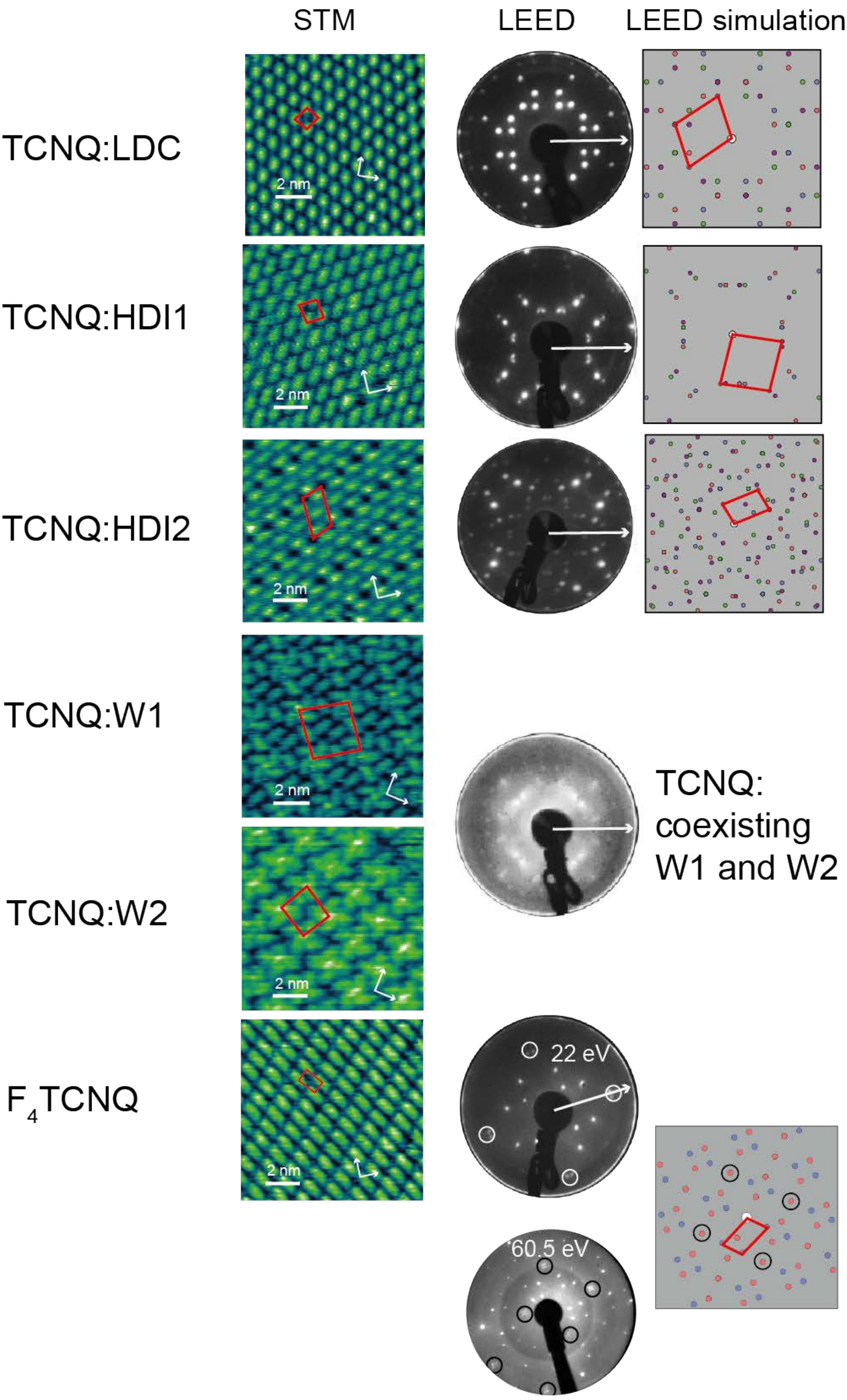
STM
LEED
LEED simulation
TCNQ:LDC
TCNQ:HDI1
TCNQ:HDI2
TCNQ:W1
TCNQ:W2
F4TCNQ
2 nm
TCNQ:
coexisting
W1 and W2
22 eV
60.5 eV

Figure S1. LEED patterns and simulated LEED patterns based on the matrices of Table S1, for the LDC, HDI1, HDI2 and, the coexisting W1 and W2 phases of TCNQ on Ag(100) and the ordered phase of F_4TCNQ on Ag(100). Electron energies are TCNQ:LDC, 18.0 eV; TCNQ:HDI1, 23.5 eV; TCNQ:HDI2, 16.0 eV; TCNQ:W1 & TCNQ:W2, 18.0 eV; F_4TCNQ, 22.0 eV and 60.5 eV. In the simulated LEED patterns diffracted beams arising from different rotational and mirror domains are shown in different colours. Unit meshes and corresponding reciprocal unit meshes are superimposed in red. <110> directions in the surface are shown as white arrows superimposed on the STM images and LEED patterns. STM tunnelling conditions (sample bias and tunnelling current): TCNQ:LDC, 0.25 V, 150 pA; TCNQ:HDI1, -0.10 V, 100 pA; TCNQ:HDI2, 1.00 V, 75 pA; TCNQ:W1, -0.05 V, 150 pA; TCNQ:W2, 1.25 V, 75 pA. F_4TCNQ, -1.00 V, 75 pA. The different conditions were chosen chosen to optimise imaging contrast.

Table S1 Summary of the different ordered adsorption phases of TCNQ and F_4TCNQ found on Ag(100).

Phase Descriptor	Matrix	Unit mesh area (Å^2)	No of molecules per unit mesh	Area per molecule (Å^2)	Preparation
TCNQ: LDC	$\begin{pmatrix} 3 & 1 \\ -2 & 3 \end{pmatrix}$	92	1	92	Low coverage deposition
TCNQ: HDI1	$\begin{pmatrix} 3.06 & 0.76 \\ 0.34 & -3.05 \end{pmatrix}$	80	1	80	Higher coverage deposition
TCNQ: HDI2	$\begin{pmatrix} 6.51 & 2.63 \\ -2.08 & 3.60 \end{pmatrix}$	240	2/3	120/80	Anneal phase HDI1 to 280-340°C
TCNQ: W1	$\begin{pmatrix} 7.84 & 5.09 \\ -6.01 & 6.22 \end{pmatrix}$	660	6	110	Anneal phase LDC to 260-340°C (mixed phases)
TCNQ:W2	$\begin{pmatrix} 2.9 & 5.7 \\ -6.1 & 2.6 \end{pmatrix}$	351	4	88	Anneal phase LDC to 340°C (mixed phases)
F_4TCNQ	$\begin{pmatrix} 4 & 2 \\ -2 & 2 \end{pmatrix}$	100	1	100	RT deposition

Table S1 summarises the main parameters of these different ordered adsorption phases. Note that the numbers of molecules per unit mesh, and hence the area per molecule, are based on the assumption that each elongated bright feature in the STM images corresponds to a molecule. For the TCNQ:HDI2 phase, this value is somewhat ambiguous; there appear to be three bright features per unit mesh but attributing all of these to TCNQ molecules seems to lead to some unreasonably short intermolecular distances. The STM image also appears to show two rather different types of bright features, one of which shows a brighter circular centre that could, perhaps, be due to the presence of an Ag adatom rather than a TCNQ molecule.

Figure S2 shows an STM image recorded from the Ag(100)-TCNQ surface under conditions leading to the formation of islands of the W2 phase, such as that outlined in purple. Notice that

the image also shows isolated and linear groups TCNQ 'windmill' structures with bright centres, possibly attributable to Ag adatoms.

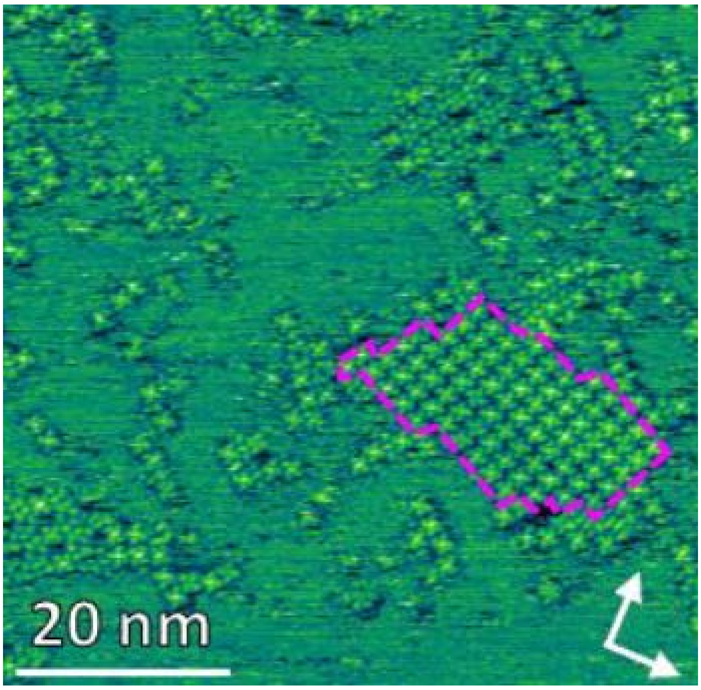

Figure S2. STM image of the Ag(100)-TCNQ surface prepared under conditions leading to the formation of islands of the W2 phase. One such island is outlined in purple. V_{sample}=1.25 V, tunnelling current 75 pA.

N 1s and F 1s SXP spectra from the TCNQ:LDC phase on Ag(100), and from the ordered phase of F_4TCNQ on Ag(100), which complement the C 1s spectra shown in Figure 2 of the main manuscript, are shown in Figure S3.

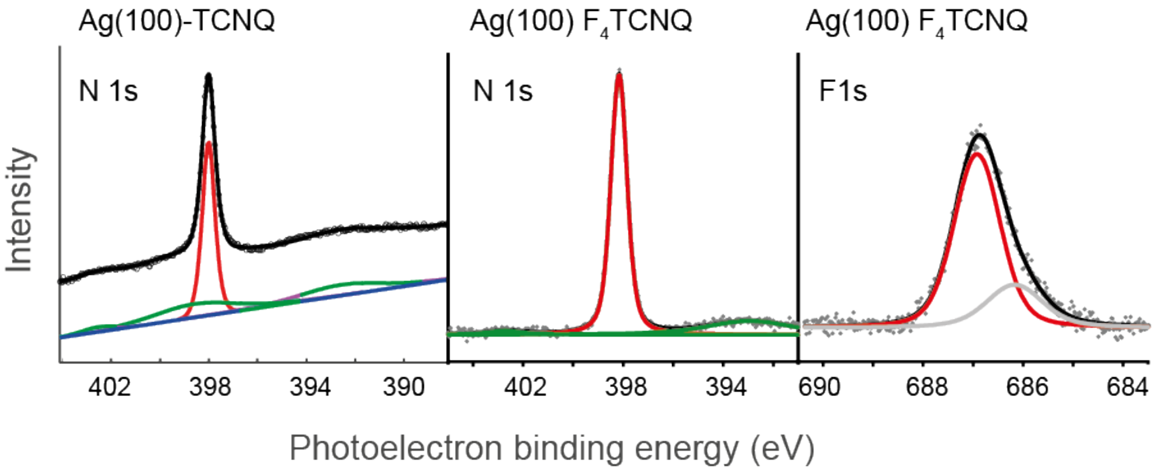

Figure S3. N 1s and F 1s SXP spectra from the LDC phase of TCNQ on Ag(100) and from the ordered phase of F_4TCNQ on Ag(100). The fits to the main peaks are shown in red, while satellites from the Ag $3d_{3/2}$ peak are shown in green. An additional F 1s component attributed to a surface species arising from radiation damage is shown in grey.

From both surfaces the N 1s spectrum is dominated by a single peak, indicating that all N atoms are in chemically similar bonding sites. The F 1s spectrum does show a second component that is attributed to the effects of radiation damage. F is known to have a particularly high cross-section for electron and photon stimulated desorption, but the presence of this component implies that some F may remain on the surface, possibly in atomic form, rather than being removed into the vacuum.

Chemical-state specific NIXSW measurements of the TCNQ:LDC and TCNQ:HDI1 phases and the ordered phase of F_4TCNQ were recorded using the C 1s, N 1s and F 1s photoemission signals as the photon energy was scanned through the normal incidence Ag(200) Bragg reflection condition, as described in the main manuscript. The experimental results and best theoretical fits are shown in Figure S4. The values of the coherent fractions and positions used in these best-fit theory curves for the TCNQ:LDC and F_4TCNQ phases (and also from the TCNQ:HCI1 phase) are reported in Table 1 of the main paper.

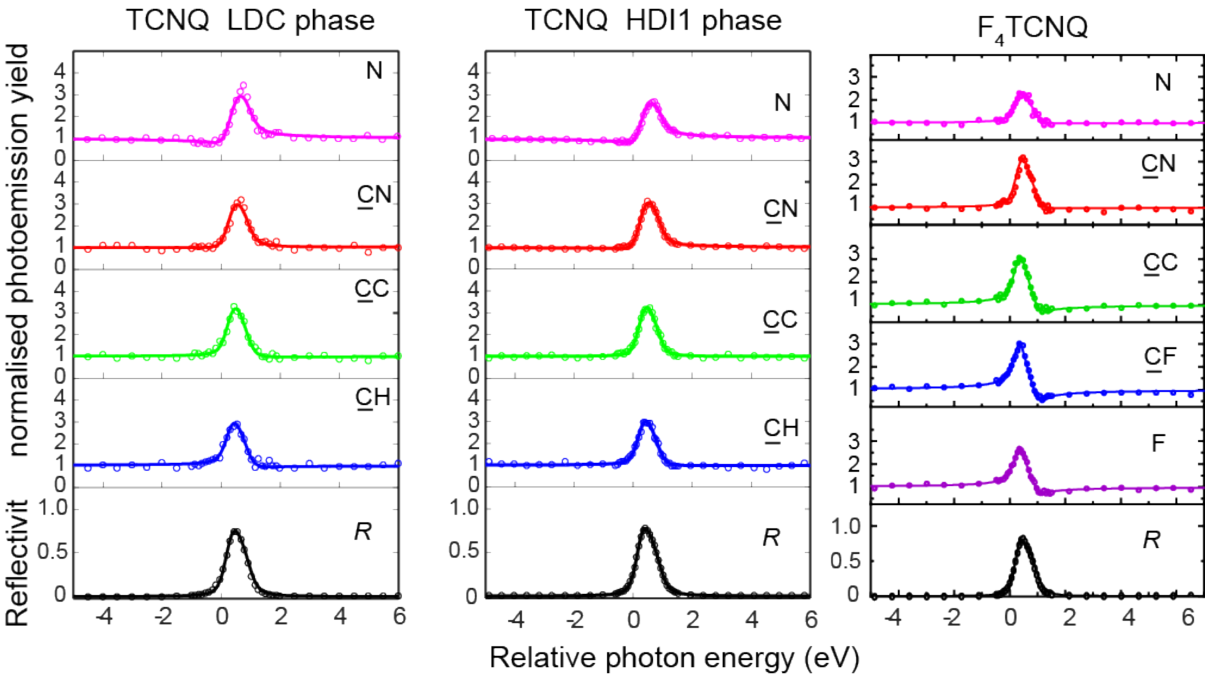

Figure S4. NIXSW photoemission yield curves obtained from samples corresponding to the TCNQ:LDC and TCNQ:HDI1 phases and the ordered phase of F_4TCNQ on Ag(100) using the (200) reflection of the substrate. Photon energies are quoted relative to the Bragg energy of approximately 3036 eV. Least square fits (solid lines) to the photoemission yields (circles) were obtained to extract the values of the coherent fractions and coherent positions reported in Table 1 of the main manuscript. Also shown is the reflectivity, *R*.

2 Additional DFT structural and energetics results: TCNQ and F_4TCNQ on Ag(100)

The results of the DFT structural optimisation for the TCNQ:LDC phase on Ag(100) at the PBE+vdW^{surf} level are shown below in Table S2 together with the experimental values. The results obtained from DFT+MBD-NL calculations of this phase are shown in Table 2 of the main paper. The two computational methods yield very similar results.

Table S2 Comparison of experimental NIXSW parameter values of the TCNQ LDC phase on Ag(100) compared with values obtained from PBE+vdW^{surf} calculations for a structural model that does not include Ag adatoms.

	TCNQ LDC Expt		TCNQ LDC DFT (no adatoms)	
Component	*f*	*D* (Å)	*f*	*D* (Å)
<u>C</u>H	0.68(10)	2.70(5)	1.00	2.76
<u>CC</u>	0.79(10)	2.65(5)	0.98	2.66
<u>C</u>N	0.70(10)	2.51(5)	1.00	2.50
N	0.81(10)	2.36(5)	1.00	2.34

The results of the structural optimisation for F_4TCNQ on Ag(100), with and without Ag adatoms, at the DFT+MBD-NL level are reported in Table 3 of the main paper, while those obtained from similar DFT+vdW^{surf} calculations are shown in Table S3.

Table S3 Comparison of experimental NIXSW parameter values of the F_4TCNQ phase on Ag(100) compared with values obtained from DFT+vdW^{surf} calculations for two alternative structural models, with and without Ag adatoms.

	F_4TCNQ – Expt.		F_4TCNQ – DFT no adatom		F_4TCNQ – DFT with adatom	
component	*f*	*D* (Å)	*f*	*D* (Å)	*f*	*D* (Å)
CF	0.79(10)	3.01(5)	1.00	2.96	0.99	2.92
CC	0.72(10)	2.94(5)	0.98	2.85	0.98	2.85
CN	0.56(10)	2.71(5)	1.00	2.56	0.96	2.77
N	0.20(10)	2.90(20)	0.99	2.28	0.69	2.74
F	0.56(10)	3.04(5)	0.97	2.94	0.97	2.95

As discussed in the main manuscript, one possible rationale for the fact that Ag adatom incorporation into the LDC phase of adsorbed TCNQ does not occur is that in this phase the high molecular packing density does not allow sufficient space. It is therefore interesting to know if Ag adatom would be favoured if adsorbed TCNQ were to adopt the larger $\begin{pmatrix} 4 & 2 \\ -2 & 2 \end{pmatrix}$ unit mesh adopted by F_4TCNQ. The results of these calculations are shown in Table S4.

Table S4 Calculated value of the adsorption energy (eV/nm2) of TCNQ, were it to be adsorbed on Ag(100) in the larger $\begin{pmatrix} 4 & 2 \\ -2 & 2 \end{pmatrix}$ unit mesh adopted by F_4TCNQ on this surface, with and without adatoms computed at the PBE+MBD-NL and PBE+vdW^{surf} level. For comparison the values obtained for adsorption in the LDC $\begin{pmatrix} 3 & 1 \\ -2 & 3 \end{pmatrix}$ unit mesh (Table 4 of the main manuscript) are included.

Unit mesh	no adatoms DFT+MBD-NL	no adatoms DFT+vdW^{surf}	with adatoms DFT+MBD-NL	with adatoms DFT+vdW^{surf}
$\begin{pmatrix} 4 & 2 \\ -2 & 2 \end{pmatrix}$	3.09	5.41	2.91	5.44
LDC $\begin{pmatrix} 3 & 1 \\ -2 & 3 \end{pmatrix}$	4.04	4.96	N/A	N/A

As remarked in the main manuscript, the STM images of the TCNQ:W2 phase do indicate that Ag adatoms may be incorporated in this phase, so DFT calculations were performed to try to cast light on this possibility. Because the TCNQ:W2 phase is incommensurate, it is not possible to perform a DFT calculation of this exact structure, but calculations using a model commensurate structure of similar size may provide some insight into the likely behaviour of the TCNQ:W2 phase. STM images of a commensurate $\begin{pmatrix} 6 & 3 \\ -3 & 6 \end{pmatrix}$ phase formed by coadsorption of TCNQ with Cs [2] are very similar to those of the W2 phase, so we choose this unit mesh for our calculation. We therefore modelled a set of TCNQ adsorption structures incorporating these molecular windmills using this $\begin{pmatrix} 6 & 3 \\ -3 & 6 \end{pmatrix}$ unit mesh, based on two alternative models, one

in which the molecular windmills are centred around an empty hollow site (see Figure S5(a)) and one in which this hollow site is occupied with an Ag adatom (Figure S5(b)).

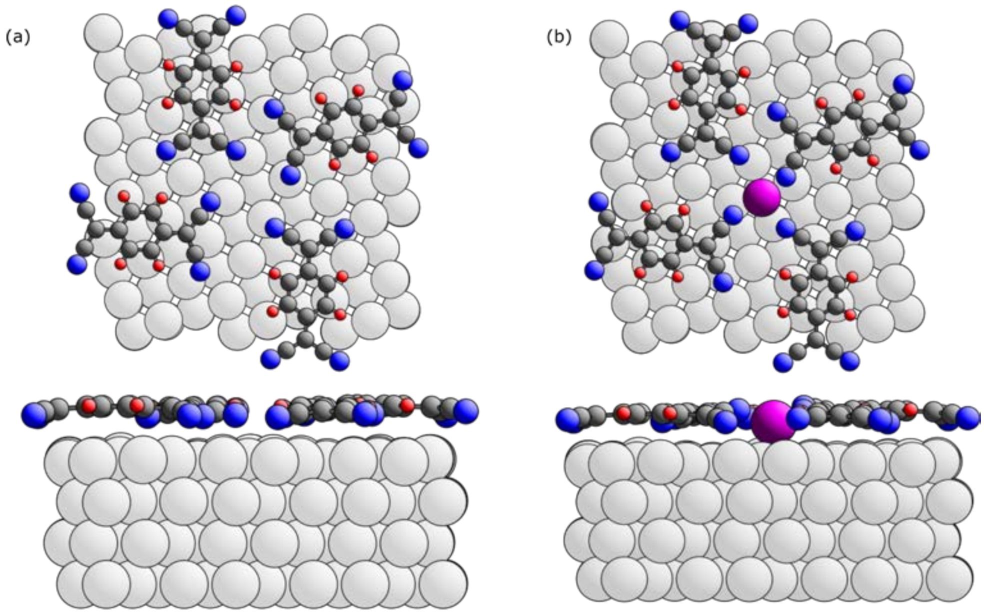

Figure S5. Top and side views of the optimised structures obtained from DFT calculations of two commensurate models of the local structure of the TCNQ:W2 phase (a) without Ag adatom (b) with an Ag adatom (coloured purple) in the centre of the molecular windmill based on the $\begin{pmatrix} 6 & 3 \\ -3 & 6 \end{pmatrix}$ unit mesh.

Table S5 Adsorption energy (eV/nm^2) of the simulated TCNQ:W2 phase 'windmill' structure on Ag(100), with and without central Ag adatoms, computed using the two different DFT methods.

Structural model	DFT+MBD-NL	DFT+vdW^{surf}
$TCNQ_4$ with adatoms	3.89	4.95
$TCNQ_4$ – no adatoms	3.83	4.90

Table S5 compares the adsorption energies of the two structural model. These do indicate an increased stability of the phase in the presence of an adatom, but the magnitude of the energy difference (5 or 6 meV) is very small; indeed, this is significantly less than *kT* at room temperature (26 meV), which would imply that structures with and without Ag adatoms would

be likely to be co-occupied, with similar probability, at room temperature. The failure of these calculations to provide clear support for adatom incorporation in the W2 phase is not particularly surprising. While modelling of an incommensurate phase by a commensurate phase may provide meaningful results on the substrate bonding of well-separated molecules, in a close-packed overlayer, differences in strain and consequential stress could well lead to significant differences in the energetics. Indeed, STM images showing ordered domains of the TCNQ:W2 phase also show isolated TCNQ 'windmills' (also apparently showing central Ag adatoms), as can be seen in Figure S2. This seems to indicate an apparent intrinsic lack of surface stress due to the formation of an ordered 2D array of these features, whereas this is a necessary component of the commensurate DFT-modelled structure. Nevertheless, it is notable that the TCNQ:W2 phase was only observed in coexistence with the TCNQ:LDC and TCNQ:W1 phases. Moreover, the STM images of the TCNQ:W1 phase unit mesh show that it contains four TCNQ in a 'windmill', surrounding a protrusion potentially interpreted as an Ag adatom, but also four additional TCNQ molecules with no evidence if an Ag adatom. It may be, therefore, that the energetic advantage of Ag adatom incorporation may be marginal, even after annealing.

References

[1] Hermann, K.E.; Van Hove, M.A. *LEEDpat (version 4.2)*, **2014**, http://www.fhi-berlin.mpg.de/KHsoftware/LEEDpat/index.html.

[2] Abdurakhmanova, N.; Floris, A.; Tseng, T.-C.; Comisso, A.; Stepanow, S.; De Vita, A.; Kern, K. Stereoselectivity and Electrostatics in Charge-Transfer Mn- and Cs-$TCNQ_4$ Networks on Ag(100), *Nat. Commun.*, **2012**, *3*, 940.
